# Simultaneous Risk-Reducing Mastectomy and Salpingo-Oophorectomy in Patients with *BRCA1* and *BRCA2* Pathogenic Variants: A Single-Center Retrospective Cohort Study

**DOI:** 10.3390/jpm16060295

**Published:** 2026-05-30

**Authors:** Raquel Diaz, Federica Murelli, Franco Alessandri, Maria Grazia Centurioni, Fabio Barra, Letizia Cuniolo, Rebecca Allievi, Abdallah Saad, Chiara Cornacchia, Francesca Depaoli, Cecilia Margarino, Ludovico Ponzielli, Elisa Bertulla, Chiara Boccardo, Ilaria Baldelli, Maria Stella Leone, Michaela Adami, Simonetta Franchelli, Marianna Pesce, Lucia Trevisan, Piero Fregatti

**Affiliations:** 1 Department of Surgical and Diagnostic Integrated Sciences—DISC, University of Genova, 16132 Genova, Italy; 2 Breast Surgery, IRCCS Ospedale Policlinico San Martino, 16132 Genova, Italy; 3 Unit of Obstetrics and Gynecology, IRCCS Ospedale Policlinico San Martino, 16132 Genova, Italy; 4 Plastic Surgery Division, IRCCS Ospedale Policlinico San Martino, 16132 Genova, Italy; 5 Department of Medical Oncology, Unit of Hereditary Cancer, IRCCS Ospedale Policlinico San Martino, 16132 Genova, Italy

**Keywords:** BRCA1, BRCA2, pathogenic variants, risk-reducing mastectomy, salpingo-oophorectomy, hereditary breast and ovarian cancer

## Abstract

**Background**: Women carrying *BRCA1* and *BRCA2* pathogenic variants face a substantially increased lifetime risk of breast and ovarian cancer. Risk-reducing bilateral mastectomy and salpingo-oophorectomy are well-established strategies to lower this risk. Traditionally, these procedures are performed in separate surgical sessions; however, a simultaneous approach may reduce the overall treatment burden. Evidence regarding the safety and feasibility of combined procedures remains limited. **Methods**: We conducted a retrospective observational study of *BRCA1* and *BRCA2* pathogenic variant carriers who underwent risk-reducing or therapeutic breast surgery at the Breast Unit of Policlinico San Martino Hospital (Genova, Italy) between January 2013 and March 2025. Patients were divided into two groups according to surgical strategy: a simultaneous procedure group undergoing risk-reducing mastectomy with immediate breast reconstruction and concurrent salpingo-oophorectomy in a single operative session, and a staged procedure group undergoing the same interventions in separate surgeries. Demographic, surgical, and postoperative variables were collected and analyzed descriptively. **Results**: A total of 124 BRCA1 and BRCA2 pathogenic variant carriers were included, with 73 patients undergoing the simultaneous approach and 51 undergoing staged procedures. The mean age was similar between the Simultaneous and Staged Procedure Groups Descriptively, similar patterns were observed across the two groups in terms of age distribution, postoperative outcomes, and length of hospital stay (mean 4.56 days). Minor complications such as seroma or delayed wound healing showed similar patterns across both groups, with no apparent increase in major complications in the simultaneous surgery group. Patients undergoing the simultaneous approach required fewer surgical sessions and were exposed to general anesthesia only once. **Conclusions**: Simultaneous risk-reducing mastectomy with immediate reconstruction and salpingo-oophorectomy appears to be a safe and feasible strategy for selected BRCA1 and BRCA2 pathogenic variant carriers. This integrated surgical approach may reduce the overall surgical burden, with descriptively similar perioperative outcome patterns.

## 1. Introduction

The management of women carrying BRCA1 and BRCA2 pathogenic variants represents one of the most delicate and complex challenges in the field of preventive oncology. These patients, often young and either planning or having recently completed their families, are faced with extremely demanding decisions from physical, psychological, and emotional perspectives [1,2]. Risk-reducing strategies, such as bilateral risk-reducing mastectomy and risk-reducing salpingo-oophorectomy, have been shown to significantly lower the incidence of breast and ovarian cancer among BRCA1 and BRCA2 pathogenic variant carriers, with residual risk levels markedly lower than those observed in the general population.

Over the past 10 to 15 years, significant progress has been made in the identification and management of individuals with hereditary cancer predisposition syndromes. Advances in next-generation sequencing have improved the speed, accuracy, and accessibility of genetic testing, allowing for earlier detection of BRCA1 and BRCA2 pathogenic variants and more timely intervention [3,4]. Simultaneously, public awareness and understanding of hereditary breast and ovarian cancer have substantially increased, particularly after actress Angelina Jolie publicly disclosed her BRCA1 pathogenic variant and risk-reducing mastectomy in 2013, an event often referred to as the “Angelina Jolie effect” According to [4,5,6], the impact of these preventive surgeries is far from negligible [7]. Mastectomy can profoundly affect body image, sexuality, and self-perception, while oophorectomy induces early surgical menopause, with possible repercussions on quality of life, cardiovascular health, bone density, and psychological well-being. Traditionally, these two interventions are planned and performed at different times, based on age, clinical priorities, and patient preferences. While this sequential approach respects the need for gradual decision-making, it entails double exposure to anesthesia, prolongs the overall surgical pathway, and subjects patients to repeated psychological stress related to surgery [8,9].

The individual oncological effectiveness of each procedure is well documented in the literature, with reported reductions in breast cancer risk by up to 95% and in ovarian cancer risk by up to 80% among patients with BRCA1 and BRCA2 pathogenic variants [10,11,12,13]. However, data regarding the simultaneous performance of these interventions during a single surgical session remain limited. Available studies primarily focus on technical aspects or short-term outcomes of individual procedures, without systematically evaluating the potential clinical, organizational, and psychological benefits of a multidisciplinary, integrated approach. This gap highlights the need to better define the true impact of a combined protocol in terms of operative safety, postoperative complications, hospital stay duration, quality of life, and patient satisfaction.

The growing implementation of personalized cancer prevention programs and multidisciplinary hereditary cancer clinics has progressively increased interest in integrated surgical strategies for *BRCA1* and *BRCA2* pathogenic variant carriers. In this setting, reducing the cumulative physical and psychological burden associated with multiple surgical procedures has become an important clinical objective. Simultaneous risk-reducing surgery may therefore represent not only a surgical alternative but also a patient-centered strategy aimed at optimizing perioperative management and improving the overall treatment pathway.

In our institution, this integrated surgical model has already been adopted as standard practice, enabling the simultaneous performance of mastectomy, immediate reconstruction, and oophorectomy in a single operation. Through a retrospective analysis of our patient cohort, we aim to assess its feasibility, safety, and overall impact.

## 2. Materials and Methods

We conducted a retrospective observational study of BRCA1 and BRCA2 pathogenic variant carriers who underwent risk-reducing or therapeutic breast surgery at the Breast Unit of Policlinico San Martino Hospital in Genova between January 2013 and March 2025. The study cohort included both patients undergoing purely risk-reducing surgery and those with a prior or concurrent diagnosis of breast cancer. All patients included in the study had confirmed pathogenic variants in either the BRCA1 or BRCA2 gene, identified through genetic testing performed as part of the institutional surveillance program for high-risk individuals. All patients were evaluated within a dedicated multidisciplinary hereditary breast and ovarian cancer pathway involving breast surgeons, gynecologists, plastic surgeons, oncologists, genetic counselors, and clinical geneticists. Surgical planning was individualized according to oncologic history, patient age, reproductive considerations, comorbidities, and patient preference.

Patients with suspected hereditary breast cancer risk undergo a dedicated genetic counseling session, during which the clinical geneticist collects a detailed personal and family medical history through targeted questions and reconstructs a comprehensive pedigree. Based on this assessment, the geneticist evaluates the appropriateness of genetic testing and, if indicated, proceeds with molecular analysis.

The study population was stratified into two cohorts based on surgical planning: those who underwent a simultaneous approach, risk-reducing mastectomy with immediate breast reconstruction and simultaneous salpingo-oophorectomy within a single operative session, and those who received the same procedures in two or more separate surgical sessions (staged approach). In the staged procedure group, not all patients had undergone salpingo-oophorectomy at the time of data collection, as surgical timing was individualized based on factors such as age, fertility considerations, and patient preference. The decision regarding surgical timing was made by a multidisciplinary team and individualized according to patient preference, clinical indication, and logistical considerations [14]. As a result, the two groups were not designed to be equivalent, and differences in baseline characteristics may reflect real-world clinical decision-making.

Patients considered suitable for the simultaneous approach generally expressed preference for reducing the number of surgical admissions and anesthetic exposures, while staged procedures were more frequently selected in patients requiring individualized oncologic timing or additional reconstructive planning.

Demographic and clinical variables were collected from patient records available in our institutional departmental database, which prospectively gathers data from all high-risk breast surgery cases. Collected variables included age at diagnosis or genetic confirmation, type of BRCA pathogenic variant (BRCA1 vs. BRCA2), smoking status (where available), history of prior surgeries, indication for surgery (purely risk-reducing vs. oncologic context), and type of reconstructive technique employed (e.g., pre-pectoral vs. submuscular implant, autologous flap, or use of tissue expanders). Due to the retrospective nature of the study, some demographic variables, including date of birth, were incomplete in a subset of patients, reflecting limitations of historical clinical records.

Surgical data included the exact nature of the breast procedure (e.g., nipple-sparing, skin-sparing mastectomy), the laterality (unilateral or bilateral), whether patients underwent salpingo-oophorectomy, and surgical access used (e.g., inframammary, vertical, radial, laparoscopic). We also recorded the type of implant or expander used, whether any previous breast surgeries were documented, the presence of adnexal pathology (such as ovarian neoplasia or atypia), and the duration of hospital stay in days following the initial procedure. In total, 124 patients were included in the analysis, with a small proportion of missing data for date of birth (25.8%) and date of surgery (1.6%).

Postoperative outcomes were assessed in terms of early complications (wound infection, hematoma, implant loss, delayed healing), need for re-intervention, and recurrence or emergence of new malignancies, when applicable. Complications were assessed during the immediate postoperative period and hospital stay, with consistent follow-up across both groups. Postoperative clinical evaluations were routinely performed by the multidisciplinary breast surgery team according to institutional follow-up protocols. When available, outpatient follow-up records were also reviewed to identify early postoperative events requiring additional clinical management.

Due to the limited granularity and heterogeneity of complication data in retrospective records, complications were analyzed descriptively and could not be consistently stratified by severity or compared quantitatively between groups. Data were analyzed descriptively. Categorical variables are reported as absolute and relative frequencies, while continuous variables are summarized using means, medians, and ranges. No inferential statistical tests were performed due to the exploratory and descriptive nature of the dataset.

Ethical review and approval were not required for this study, as it is a retrospective analysis based on anonymized data collected during routine clinical practice. In accordance with the General Data Protection Regulation (Regulation (EU) 2016/679) and applicable national and institutional policies governing observational studies, research not involving prospective intervention or identifiable data may not require formal Ethics Committee approval. All patients provided informed consent for the use of anonymized data for research purposes.

## 3. Results

Out of the total cohort of 124 BRCA1 and BRCA2 pathogenic variant carriers evaluated between January 2013 and March 2025, all patients were biologically female and underwent risk-reducing or therapeutic breast surgery. Patients were stratified into two groups based on the surgical timing of risk-reducing procedures. The main demographic, clinical, and surgical characteristics of the study population are summarized below to provide a structured overview of the two groups (Table 1).

A subset of patients underwent a coordinated surgical protocol that included mastectomy, immediate breast reconstruction, and risk-reducing salpingo-oophorectomy performed during a single operative session. These patients were classified as the Simultaneous Procedure Group. The remaining patients underwent the same set of interventions in multiple, separate surgical sessions and were classified as the Staged Procedure Group.

A total of 73 patients were included in the Simultaneous Procedure Group, while 51 patients belonged to the Staged Procedure Group. The main demographic and clinical characteristics of the two groups are summarized in Table 1. Descriptively, the two groups showed partially different baseline clinical characteristics, likely reflecting real-world surgical selection and individualized multidisciplinary decision-making. A relevant proportion of patients had a history of breast cancer and previous breast surgery, reflecting the inclusion of both risk-reducing and therapeutic cases within the cohort. Surgical indication and reconstructive strategies were therefore influenced by both oncologic and preventive considerations. However, due to the non-randomized design, formal comparability between groups cannot be assumed.

The mean age was similar between the Simultaneous and Staged Procedure Groups, as summarized in Table 1. Notably, 32 patients (25.8%) had missing or incomplete date-of-birth data and were excluded from age-based analyses.

This was mainly due to incomplete historical records in the institutional database, particularly for patients treated in earlier years. Importantly, other key clinical and surgical variables were complete and available for analysis.

Regarding pathogenic variant status, 54.0% of patients (n = 67) carried a BRCA1 pathogenic variant, while the remaining 46.0% (n = 57) had a BRCA2 pathogenic variant. The distribution of BRCA1 and BRCA2 pathogenic variants was similar between the Simultaneous and Staged groups.

The majority of surgical procedures involved bilateral nipple-sparing or skin-sparing mastectomies, commonly performed through inframammary (33.9%) or vertical incisions. Reconstructive techniques varied, with tissue expanders used in 55.6% of cases, followed by direct-to-implant reconstruction or other methods. In the Simultaneous Procedure Group, definitive implant-based reconstruction was more frequently performed, whereas tissue expanders were more commonly used in the Staged Procedure Group, reflecting individualized reconstructive planning based on oncologic and anatomical considerations.

**Figure 1:**
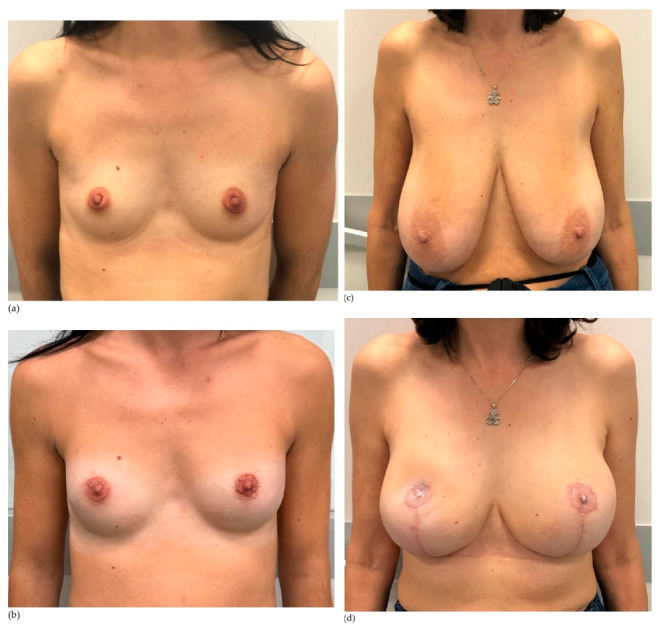
Representative clinical outcomes following bilateral risk-reducing mastectomy with immediate definitive implant-based breast reconstruction in BRCA1/BRCA2 pathogenic variant carriers undergoing simultaneous surgery. (**a**,**b**) Preoperative and postoperative views following bilateral nipple-sparing mastectomy with retromuscular definitive implant reconstruction. (**c**,**d**) Preoperative and postoperative views following skin-reducing nipple-sparing mastectomy with retromuscular definitive implant reconstruction.

The choice of reconstructive technique was individualized based on patient characteristics, prior treatments, and anatomical considerations, reflecting real-world multidisciplinary decision-making. Representative postoperative reconstructive outcomes following simultaneous risk-reducing surgery are shown in Figure 1.

Previous breast surgery, often related to prior breast cancer treatment, was reported in 42.7% of patients. Salpingo-oophorectomy was performed in all patients in the simultaneous group, while in the staged group only a subset of patients had undergone the procedure at the time of analysis. Among these, only a small minority (5.6%) presented with histopathological evidence of ovarian neoplasia or atypia. The majority of adnexal surgeries were performed as planned, risk-reducing procedures.

Postoperative hospital stay was consistent across the cohort, with a mean duration of 4.56 days and a median of 4 days (range: 3–13 days). Descriptively, length of hospital stay appeared similar across the Simultaneous and Staged groups. Despite the potentially longer duration of a single combined operation, the Simultaneous group did not require prolonged hospitalization.

Postoperative complication rates were assessed descriptively due to the retrospective nature of data collection. Observed postoperative complications mainly included minor wound dehiscence, seroma formation, delayed wound healing, and isolated cases of infection requiring surgical revision. Major complications requiring reoperation were rare in both groups.

Descriptively, both groups showed similar patterns of minor complications, including seroma, delayed healing, or wound dehiscence, with no apparent differences in major complications such as implant loss, reoperation, or severe infections (Table 2). A detailed quantitative stratification of complications by severity and group was not feasible due to the retrospective nature of data collection. Importantly, the integration of breast and gynecologic surgical teams in the Simultaneous Procedure Group did not lead to an increased rate of adverse outcomes.

In addition to clinical outcomes, patients in the Simultaneous Procedure Group underwent fewer surgical sessions, with each patient exposed to general anesthesia only once.

This translated into a shorter overall recovery process, and fewer cumulative admissions, and may suggest potential advantages from a patient perspective. Although these outcomes were not formally assessed in the present study, the reduction in the number of surgical procedures and hospital admissions may suggest potential organizational and patient-centered advantages.

In summary, descriptively, no clear differences were observed between the Simultaneous and Staged groups in terms of age, hospital stay, or postoperative complications, while the Simultaneous Procedure Group underwent fewer surgical sessions.

## 4. Discussion

Risk-reducing surgery plays a pivotal role in the management of women carrying BRCA1 and BRCA2 pathogenic variants [15,16,17]. In these patients, risk-reducing mastectomy and salpingo-oophorectomy are well-established interventions capable of significantly lowering the risk of breast and ovarian cancer [18]. However, the optimal timing and surgical planning of these procedures remain an area of ongoing discussion, particularly regarding whether they should be performed sequentially or within a single operative session. Combining risk-reducing surgical procedures may reduce the overall treatment burden while maintaining oncologic safety. Reported complication rates following risk-reducing mastectomy in the literature generally range between 15% and 20%, depending on patient selection and surgical technique. In our cohort, complication patterns were consistent with these ranges when assessed descriptively. The role of nipple-sparing mastectomy in BRCA mutation carriers remains a topic of ongoing debate. While its oncologic safety has been increasingly supported in selected patients, long-term data in high-risk populations are still limited. Careful patient selection and multidisciplinary evaluation are therefore essential when considering this approach.

Although the available literature remains limited, some studies have explored the feasibility of combining risk-reducing breast and gynecologic procedures. Perabò et al. described a novel surgical technique in which risk-reducing mastectomy with immediate reconstruction was combined with laparoscopic salpingo-oophorectomy through a single access route, demonstrating the technical feasibility of performing these procedures simultaneously [19]. More recently, Saccardi et al. reported encouraging results in BRCA1 and BRCA2 pathogenic variant carriers undergoing combined risk-reducing surgery, highlighting not only the safety of the approach but also high levels of patient satisfaction when adequate multidisciplinary planning and counseling were provided [20].

In this context, surveillance strategies, including regular mammography and breast MRI, represent a valid alternative to risk-reducing surgery in selected patients. In our clinical practice, all patients undergo multidisciplinary counseling, including evaluation by a clinical geneticist, during which different management options—including surveillance and surgical approaches—are discussed in detail, although these aspects were not systematically captured in the present dataset.

Within this evolving context, the integration of breast and gynecologic procedures into a single operative session may offer several potential advantages. From a clinical perspective, the simultaneous approach allows patients to undergo both preventive interventions during a single exposure to general anesthesia, potentially reducing cumulative surgical stress and shortening the overall treatment pathway. This aspect may be particularly relevant for young women with hereditary cancer risk, who often face complex preventive decisions while balancing family, professional, and personal considerations.

In addition to patient-related benefits, coordinated surgical strategies may also have important organizational implications. Consolidating procedures into a single hospitalization can simplify perioperative management and optimize the use of healthcare resources, particularly in high-volume breast units managing increasing numbers of genetically high-risk individuals. Effective collaboration between breast surgeons, gynecologic surgeons, anesthesiologists, and genetic counselors is essential to ensure appropriate patient selection and to maintain high standards of surgical safety. Reconstructive decision-making in this setting is inherently individualized and based on multiple factors, including patient comorbidities, prior oncologic treatments, anatomical characteristics, and patient preference. In our cohort, the variability in reconstructive techniques reflects real-world multidisciplinary decision-making rather than a standardized protocol. These aspects are particularly relevant in high-risk patients, where both oncologic safety and reconstructive outcomes must be carefully balanced.

Beyond the surgical aspects, the psychological dimension of preventive surgery should not be underestimated [7]. Women carrying BRCA1 and BRCA2 pathogenic variants often experience significant emotional and decisional burden related to cancer risk and risk-reducing interventions. The possibility of addressing multiple preventive procedures within a single surgical pathway may help reduce repeated exposure to surgical stress and contribute to a more cohesive and manageable treatment experience.

Overall, our findings suggest that coordinated risk-reducing surgery can be safely implemented in carefully selected patients. Within this framework, an integrated simultaneous approach may represent a valuable strategy that aligns with the principles of modern multidisciplinary and patient-centered oncologic care.

From a practical perspective, the simultaneous surgical approach may represent a valuable organizational strategy in specialized hereditary cancer centers. Reducing the number of surgical admissions and anesthetic exposures may simplify perioperative management and potentially improve the overall patient experience. Furthermore, coordinated multidisciplinary pathways involving breast surgeons, gynecologists, plastic surgeons, and genetic counselors may facilitate more integrated and patient-centered care for *BRCA1* and *BRCA2* pathogenic variant carriers undergoing preventive surgery.

Future prospective multicenter studies with standardized perioperative and complication reporting are needed to better define the role of simultaneous risk-reducing surgery in *BRCA1* and *BRCA2* pathogenic variant carriers. In particular, future research should investigate patient-reported outcomes, quality of life, healthcare resource utilization, and long-term oncologic safety. Further evaluation of patient satisfaction and psychological impact may also help optimize multidisciplinary preventive surgical pathways in hereditary cancer care.

This study has several limitations. First, the relatively small sample size may have limited the statistical power to detect significant differences between the simultaneous and staged surgical approaches. Second, the non-randomized allocation of patients introduces a potential risk of selection bias, as surgical planning was influenced by clinical evaluation, logistical factors, and patient preference, potentially resulting in non-equivalent groups. Therefore, direct comparisons between groups should be interpreted with caution. Third, the retrospective design limited the completeness of available data, particularly regarding postoperative complications and long-term outcomes such as recurrence and patient-reported measures. Age likely played a key role in determining the timing of salpingo-oophorectomy, as younger patients may delay the procedure to preserve ovarian function and fertility. However, this aspect could not be fully explored due to missing age data. Missing demographic data, particularly date of birth, represent a limitation of this retrospective analysis and restricted the possibility of performing reliable age-based comparisons between groups. The lack of structured complication data also limited the ability to provide detailed quantitative comparisons of postoperative outcomes between groups. Furthermore, the heterogeneity of the cohort, including both risk-reducing and therapeutic cases, different reconstructive techniques, and patients with prior surgeries, may have influenced the observed outcomes. Finally, key intraoperative variables such as operative time, anesthesia duration, and intraoperative outcomes were not consistently available and could not be analyzed. The purely descriptive nature of the analysis also limits the interpretability of the findings. Information regarding the presence of occult breast cancer in mastectomy specimens was not consistently available and could not be analyzed, representing an additional limitation of this study. Finally, the single-center nature of the study may limit the generalizability of the findings, as all procedures were performed in a high-volume specialized center.

## 5. Conclusions

The simultaneous surgical approach combining risk-reducing mastectomy, immediate breast reconstruction, and salpingo-oophorectomy appears to be a safe and feasible strategy in a real-world heterogeneous population of BRCA1 and BRCA2 pathogenic variant carriers. Our findings support the potential value of coordinated multidisciplinary surgery in the management of high-risk patients. Further prospective multicenter studies are warranted to confirm these results and to better define the role of this strategy within risk-reducing surgical pathways.

## Figures and Tables

**Table 1:** Baseline demographic and clinical characteristics of patients undergoing simultaneous or staged procedures.

Characteristics	Simultaneous Group (n = 73)	Staged Group (n = 51)
Mean age, years	52.5	54.8
Median age, years	52	54
BRCA1 pathogenic variant, n (%)	35 (48.0)	32 (62.7)
BRCA2 pathogenic variant, n (%)	38 (52.0)	19 (37.3)
Previous breast surgery, n (%)	20 (27.4)	28 (55.0)
Risk-reducing surgery, n (%)	50 (68.4)	23 (45.1)
Therapeutic surgery for cancer, n (%)	23 (31.6)	28 (54.9)
Prosthetic reconstruction, n (%)	40 (54.8)	15 (29.4)
Expander reconstruction, n (%)	33 (45.2)	36 (70.6)

**Table 2:** Postoperative outcomes in the simultaneous and staged procedure groups.

Outcome	Simultaneous Group (n = 73)	Staged Group (n = 51)
Mean hospital stay, days	4.5	4.6
Overall postoperative complications, n (%)	5 (6.8%)	4 (7.8%)
Major complications requiring reoperation, n (%)	1 (1.4%)	1 (2.0%)

## Data Availability

The data supporting the findings of this study are derived from institutional clinical records and are not publicly available due to privacy and ethical restrictions. The data presented in this study are available on reasonable request from the corresponding author.
